# AI Decision-Making Performance in Maternal–Fetal Medicine: Comparison of ChatGPT-4, Gemini, and Human Specialists in a Cross-Sectional Case-Based Study

**DOI:** 10.3390/jcm15010117

**Published:** 2025-12-24

**Authors:** Matan Friedman, Amit Slouk, Noa Gonen, Laura Guzy, Yael Ganor Paz, Kira Nahum Sacks, Amihai Rottenstreich, Eran Weiner, Ohad Gluck, Ilia Kleiner

**Affiliations:** 1 Department of Obstetrics and Gynecology, E. Wolfson Medical Center, Holon 5822012, Israeleranw@wmc.gov.il (E.W.); kleiner.ilia@gmail.com (I.K.); 2 Gray Faculty of Medical & Health Sciences, Tel Aviv University, Tel Aviv 6997801, Israel

**Keywords:** artificial intelligence, clinical decision-making, maternal–fetal medicine, obstetrics, Large Language Models

## Abstract

**Background/Objectives:** Large Language Models (LLMs), including ChatGPT-4 and Gemini, are increasingly incorporated into clinical care; however, their reliability within maternal–fetal medicine (MFM), a high-risk field in which diagnostic and management errors may affect both the pregnant patient and the fetus, remains uncertain. Evaluating the alignment of AI-generated case management recommendations with those of MFM specialists, emphasizing accuracy, agreement, and clinical relevancy. Study Design and Setting: Cross-sectional study with blinded online evaluation (November–December 2024); evaluators were blinded to responder identity (AI vs. human), and case order and response labels were randomized for each evaluator using a computer-generated sequence to reduce order and identification bias. **Methods:** Twenty hypothetical MFM cases were constructed, allowing standardized presentation of complex scenarios without patient-identifiable data and enabling consistent comparison of AI-generated and human specialist recommendations. Responses were generated by ChatGPT-4, Gemini, and three MFM specialists, then assessed by 22 blinded board-certified MFM evaluators using a 10-point Likert scale. Agreement was measured with Spearman’s rho (ρ) and Cohen’s (κ); accuracy differences were measured with Wilcoxon signed-rank tests. **Results:** ChatGPT-4 exhibited moderate alignment (mean 6.6 ± 2.95; ρ = 0.408; κ = 0.232, *p* < 0.001), performing well in routine, guideline-driven scenarios (e.g., term oligohydramnios, well-controlled gestational hypertension, GDMA1). Gemini scored 7.0 ± 2.64, demonstrating effectively no consistent inter-rater agreement (κ = −0.024, *p* = 0.352), indicating that although mean scores were slightly higher, evaluators varied widely in how they judged individual Gemini responses. No significant difference was found between ChatGPT-4 and clinicians in median accuracy scores (Wilcoxon *p* = 0.18), while Gemini showed significantly lower accuracy (*p* < 0.01). Model performance varied primarily by case complexity: agreement was higher in straightforward, guideline-based scenarios and more variable in complex cases, whereas no consistent pattern was observed by gestational age or specific clinical domain across the 20 cases. **Conclusions:** AI shows promise in routine MFM decision-making but remains constrained in complex cases, where models sometimes under-prioritize maternal–fetal risk trade-offs or incompletely address alternative management pathways, warranting cautious integration into clinical practice. Generalizability is limited by the small number of simulated cases and the use of hypothetical vignettes rather than real-world clinical encounters.

## 1. Introduction

In recent years, the integration of artificial intelligence (AI) in healthcare has grown exponentially, with AI models, such as ChatGPT-4, increasingly used to generate responses to medical inquiries [1,2] as well as successfully pass medical tests, such as the United States Medical Licensing Examination (USMLE) [3]. These advancements hold promise for enhancing patient education, expanding access to medical information, and reducing the burden on healthcare providers. However, as AI-generated responses become more sophisticated [4,5], it is crucial to assess their reliability, accuracy, and perceived authenticity, especially in sensitive fields like gynecology, where patients require specialized knowledge and nuanced communication [6,7].

Studies have shown that AI tools can aid in diagnosing, interpreting complex data, and managing patient inquiries, showing high capabilities, yet questions remain regarding their role in delivering patient-specific, clinically accurate information and in decision- making [8,9]. Recent SWOT-based evaluations across clinical medicine also emphasize that, despite rapid progress in diagnostics, therapeutics, and safety, the overall clinical impact of AI still requires rigorous specialty-level evaluation to ensure safe and appropriate integration [10]. The field of obstetrics and gynecology, and more specifically maternal–fetal medicine, entails patient concerns that involve highly technical aspects of treatment and prognosis, including areas that benefit from personalized and source-dependent information responses by specialists, which is not always the case when using AI-provided responses [11,12,13]. Furthermore, the current widespread utilization of FAQ online forums allows users to reach out to caregivers more easily and obtain information without necessarily reaching these specialists [14].

This study aims to evaluate whether AI-generated case management decisions in standardized, clinically realistic scenarios are distinguishable from those provided by trained specialists when reviewed by other physicians in the field. Our primary goal is to understand the potential of AI tools to provide well-explained case management decisions that are clinically accurate and aligned with professional standards. Despite rapid adoption, no study has quantified whether LLM recommendations concur with expert MFM judgment. We hypothesized that AI models would demonstrate at least moderate agreement with specialists in routine cases but lower agreement in complex scenarios. Case complexity was pre-classified a priori as straightforward when guideline-directed management was clear and widely accepted and complex when multiple management strategies or significant controversies existed (e.g., twin–twin transfusion syndrome, placenta accreta, alloimmunization). This research aims to add to the growing body of literature on AI’s integration in healthcare, focusing on the accuracy and contextual relevance of AI-generated decisions in maternal–fetal medicine. For this study, a response was considered clinically accurate if evaluators judged it to be consistent with current evidence-based guidelines or standard MFM practice, and well-explained if the rationale was clear, logically structured, and explicitly linked to the clinical features presented in the case. Because real patient outcomes were not available, this study was designed to evaluate perceived alignment and reasoning consistency rather than diagnostic accuracy. This approach isolates cognitive and decision-making concordance between specialists and AI, providing insight into the models’ potential as supportive rather than autonomous tools.

## 2. Materials and Methods

### 2.1. Study Design and Participants

For this study, an online cross-sectional study was conducted between November and December 2024.

### 2.2. Scenario Development

Twenty hypothetical maternal–fetal medicine scenarios were generated based on frequently encountered cases in outpatient clinics and inpatient units. Draft scenarios were derived from anonymized real-world cases and then iteratively refined. Subsequently, eight independent board-certified MFM specialists reviewed all scenarios for clinical realism, clarity, and concordance with current guidelines, and any discrepancies were resolved by consensus to formulate the final version. Cases were hypothetical and fully de-identified to ensure no patient information was used. Responders were asked to provide a clear case management suggestion alongside a short explanation to the suggested management (up to 3 sentences). ChatGPT-4 and Gemini version exp-1121 were compared with three fellowship-trained maternal–fetal medicine specialists, each with ≥10 years of independent practice and no prior collaboration with AI systems. The sample size of three experts was chosen to maintain methodological feasibility while ensuring senior clinical representation, reflecting common practice in vignette-based comparison studies where a small, highly experienced reference group is used to represent expert standard-of-care decision-making. Each practicing maternal–fetal medicine Specialist, who completed a recognized maternal–fetal medicine fellowship in the US, Canada, or Europe, was allowed adequate time to respond to the scenario without using references to obtain answers. The physicians were blinded to the study design and unaware that their responses would be compared to chatbot responses. The three clinicians were purposively sampled: fellowship-trained, ≥10 years’ practice, no prior AI collaboration. ChatGPT and Gemini responses were generated by inserting the question into a fresh session in November 2024. This study followed relevant elements of the STROBE reporting guideline for cross-sectional studies, adapted to a hypothetical vignette-based design. Scenarios were generated to match known cases from daily work in maternal–fetal medicine clinics or departments, de-identifying any information by removing unique identifiers to ensure they were HIPAA-compliant. The E. Wolfson Medical Center Institutional Review Board deemed the study exempt (category 4) because it used de-identified hypothetical cases and posed no risk to human subjects.

### 2.3. Procedures

**Figure 1:**
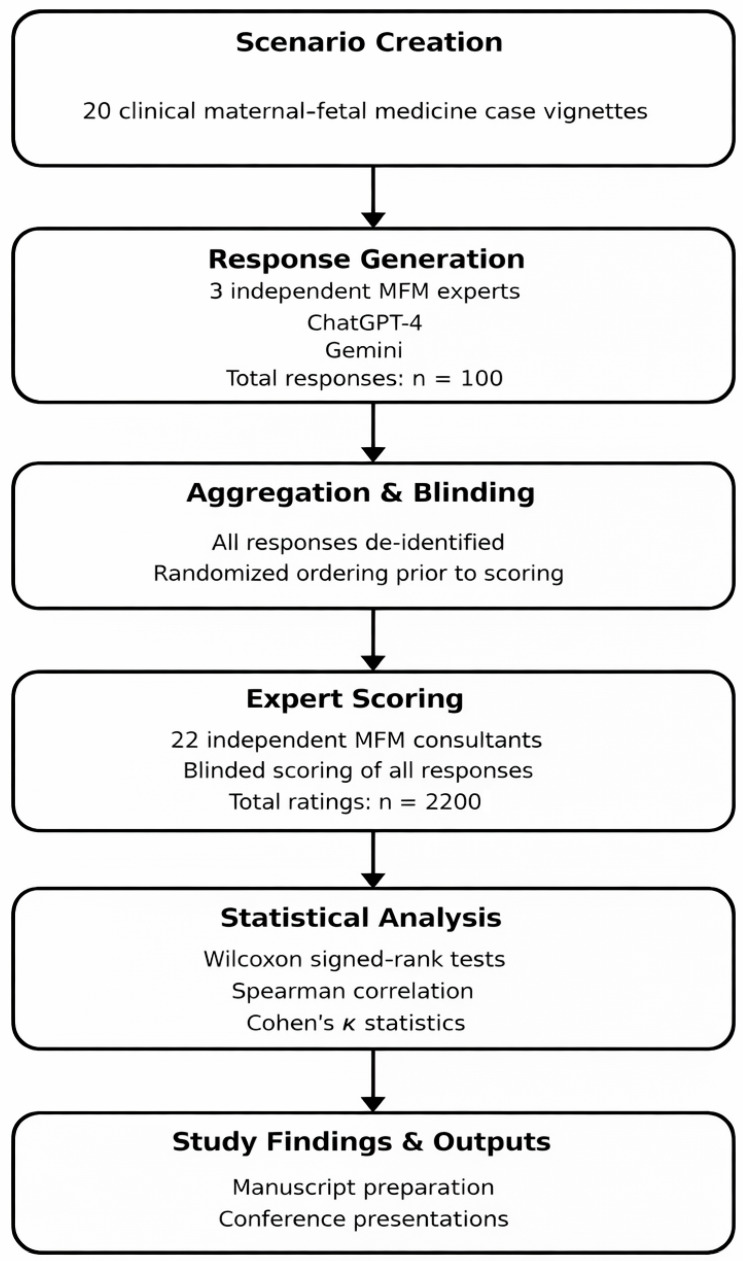
CONSORT-style workflow outlining case generation, response acquisition, and blinded evaluation process.

This study was based on four main phases, as outlined in Figure 1. The first phase involved the creation and formulation of 20 case scenarios (Supplementary S1). The cases were developed based on frequently arising cases in the daily work of maternal–fetal medicine clinics and departments. Subsequently, eight independent physicians, board-certified maternal–fetal medicine specialists, reviewed the proposed scenarios and formulated the final version, resolving discrepancies through group discussion until consensus was reached; when agreement could not be achieved, a senior MFM specialist adjudicated the final wording.

No additional pilot testing beyond expert review was performed

During the second phase of this study, answer collection took place. Researchers accessed OpenAI (ChatGPT version 4 and Gemini) and posted the 20 cases using a standardized format, using a prompt requesting the answer as a seasoned MFM specialist using evidence-based medicine decision-making. ChatGPT and Gemini were asked to provide a clear case management and explicitly asked for the basis of the response in up to three sentences. For each scenario, ChatGPT-4 and Gemini were queried once in a fresh session (18–22 November 2024), and the first complete response was recorded to avoid selection of favored outputs; conversations were cleared between entries to prevent context carry-over. Researchers recorded responses. Additionally, three independent maternal–fetal medicine specialists were given the same instructions, and their responses were recorded.

In the third phase, an evaluation of responses was undertaken. Physicians’ answers and ChatGPT and Gemini’s answers were randomly arranged while keeping identifiable information concealed (e.g., omitting statements such as “I’m an artificial intelligence”) and randomly ordered, stripped of revealing information, and labeled response 1 or response 2, 3, and 4; thus, evaluators were blinded to whether each recommendation originated from an AI model or a human specialist, and to the specific identity of the responder. Case order and assignment of response labels (1–4) were generated using a computer-based randomization algorithm in Excel, and the same randomized order was applied for all evaluators. We then asked 30 independent practicing maternal–fetal medicine specialists working in different hospitals to assess their agreement with the different case management suggested, evaluating each answer’s accuracy, and judging how closely each response aligns with their clinical recommendations. Case evaluations were grounded in contemporary local and international maternal–fetal medicine guidelines, including recommendations from the International Society for the Study of Hypertension in Pregnancy (ISSHP), the American College of Obstetricians and Gynecologists (ACOG), and the Royal College of Obstetricians and Gynaecologists (RCOG), and local guidelines as applicable to the clinical domain of each case.

Overall, 22 completed the assessment, and each evaluator assessed 5 recommended managements per case, evaluating all 20 cases, with 100 recommended managements all altogether, providing adequate precision for non-parametric agreement estimates. All five responses (three clinicians plus ChatGPT-4 and Gemini) were obtained and evaluated for each of the 20 scenarios; there were no missing responses.

Later, each case was classified a priori as either straightforward (e.g., oligohydramnios at term, well-controlled gestational hypertension, GDMA1) or complex (e.g., twin–twin transfusion syndrome, placenta accreta, alloimmunization with anti-Kell). Classification was based on whether guideline-directed management was available (straightforward) or multiple management strategies/controversies existed (complex). Evaluators were blinded to responder identity (AI vs. human). All responses were stripped of stylistic cues or AI identifiers and presented in uniform format. Blinding was prioritized to reduce halo bias, accepting the trade-off of limited contextual information. Evaluators were asked to rate their agreement with the suggested management on a scale from 1 to 10 (1-do not agree to 10-strongly agree). All evaluators scored independently; the median of 22 ratings per response was used for analyses.

Definition of metrics:

- Agreement score—median evaluator Likert rating (1 = do not agree; 10 = strongly agree).
- Accuracy score—evaluators’ judgment of evidence-based correctness of each response, treated as a secondary outcome variable analyzed alongside agreement scores.
- Correlation (ρ)—Spearman’s coefficient comparing AI vs. clinician ratings.
- Cohen’s κ—inter-rater reliability among evaluators, where κ = 0.21–0.40 represents fair agreement.

In the fourth phase, a comprehensive assessment of responses was performed through the planned statistical analyses (agreement scores, correlations, and inter-rater reliability). ChatGPT and Gemini’s performance were assessed and compared with the human physicians’ recommended management. The study focused on the agreement between AI-generated and specialist recommendations rather than outcome-based accuracy.

## 3. Statistical Analysis

To analyze the data from this study, we employed methods appropriate for paired and categorical data to evaluate agreement and quality ratings. In this manuscript, “agreement score” refers to the median evaluator Likert rating (1–10) for each response, “inter-rater agreement” refers to Cohen’s κ among evaluators, and “concordance” refers to Spearman’s correlation (ρ) between AI and clinician scores. First, the Wilcoxon signed-rank tests compared paired evaluator-level scores (ChatGPT-4 vs. clinician median, Gemini vs. clinician median) across all cases, treating each evaluator–case pair as one observation. This non-parametric test is suitable given the ordinal nature of the agreement and quality scales and that it does not assume a normal distribution of the scores. Second, Cohen’s kappa statistic was employed to assess the level of agreement between evaluators when judging the accuracy and quality of the responses. This test measures inter-rater reliability, accounting for agreement occurring by chance. These statistical tools allow for a robust evaluation of AI’s alignment with clinical recommendations and the variability among evaluators, offering insights into the consistency and reliability of AI in maternal–fetal management scenarios. All analyses were two-tailed with significance set at *p* < 0.05. Cohen’s κ values were interpreted using Landis & Koch criteria (κ = 0.21–0.40 = fair; 0.41–0.60 = moderate). Spearman’s rank-order correlation (ρ) was used to assess case-level concordance between AI and clinician ratings because the underlying Likert scores are ordinal and may not follow a normal distribution.

The primary outcome was the degree of evaluators’ agreement with the different statements. Secondary outcomes were performed to identify specific areas with enhanced alignment between AI tools and physicians.

Statistical analysis was performed by SPSS version 30.0 (IBM Corp., Armonk, NY, USA). Continuous variables are presented as mean ± standard deviation (SD).

## 4. Results

We invited 30 maternal–fetal medicine specialists to participate; 22 completed all evaluations (73% response rate). Each evaluator reviewed all 100 management recommendations, requiring approximately 40 min. Once complete, their evaluation form was submitted online.

**Figure 2:**
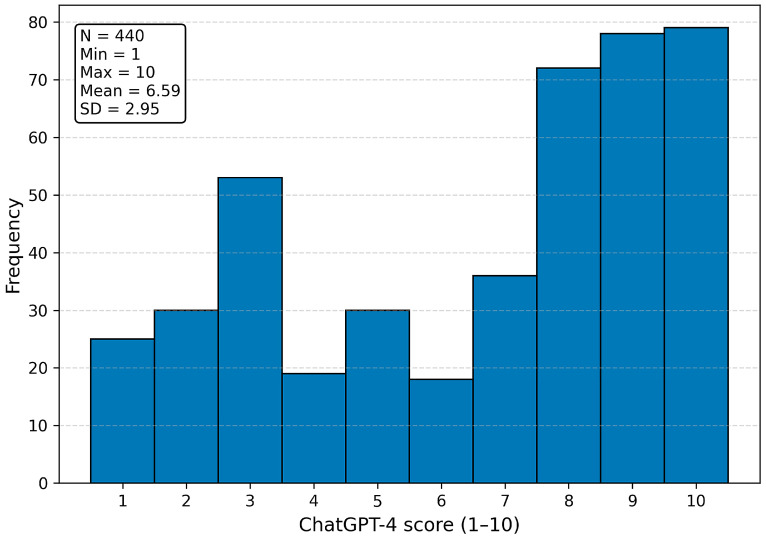
Distribution of ChatGPT-4 agreement/accuracy scores across all evaluated responses (N = 440 evaluator–case ratings). Bars represent the frequency of evaluator Likert scores (1–10) assigned to ChatGPT-4 recommendations across 20 clinical scenarios. A statistics panel displays the minimum, maximum, mean, and standard deviation of scores.

**Figure 3:**
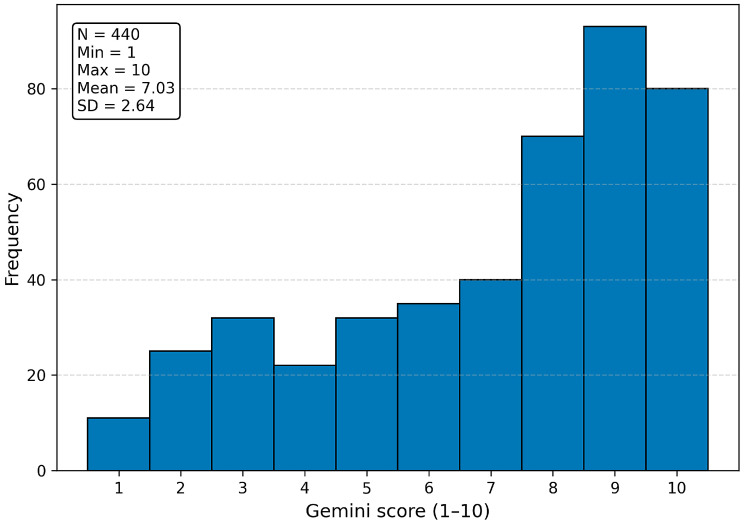
Distribution of Gemini agreement/accuracy scores across all evaluated responses (N = 440 evaluator–case ratings). Bars represent the frequency of evaluator Likert scores (1–10) assigned to Gemini recommendations across 20 clinical scenarios. A statistics panel displays the minimum, maximum, mean, and standard deviation of scores.

**Figure 4:**
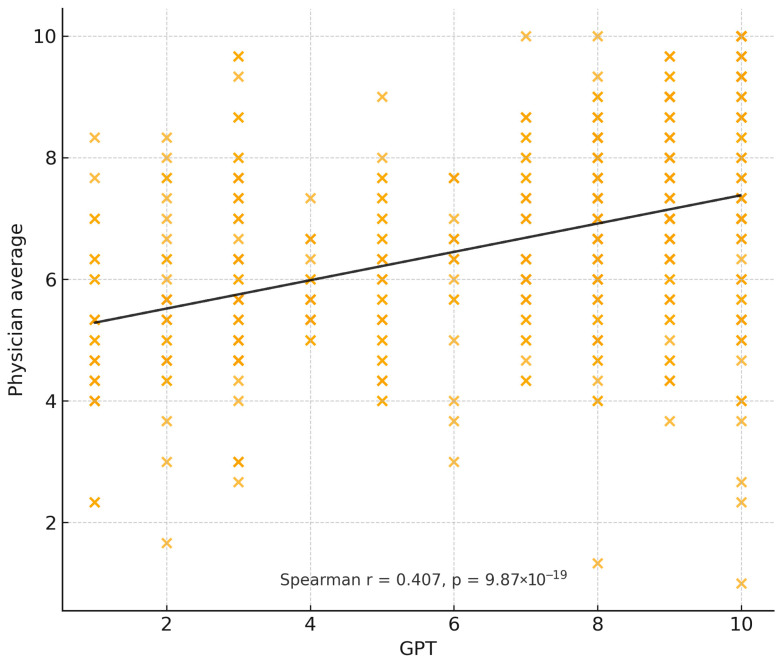
Relationship between ChatGPT-4 recommendation scores and mean clinician scores across all evaluator–case pairs. Each point represents a single evaluator’s rating for a given case. A linear trendline (dashed) is shown for visualization. Spearman correlation (ρ) and *p*-value quantify the strength of association between ChatGPT-4 and specialist evaluations.

**Figure 5:**
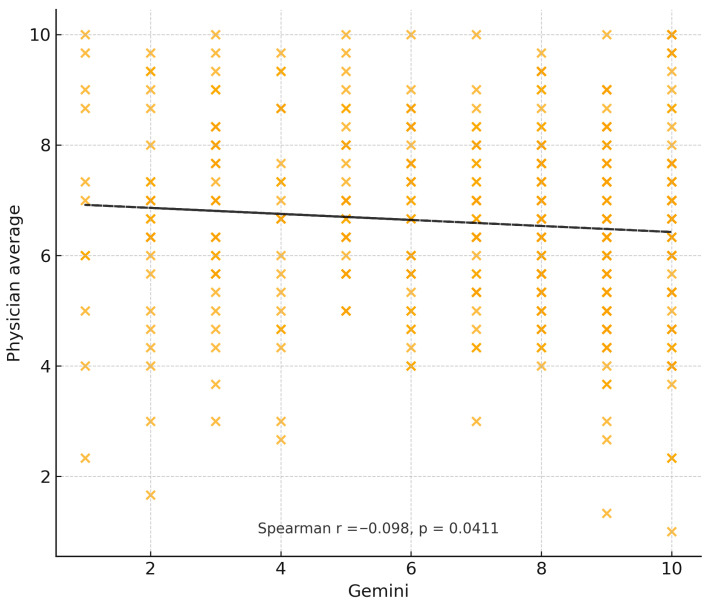
Relationship between Gemini recommendation scores and mean clinician scores across all evaluator–case pairs. Each point reflects an evaluator’s rating for a single scenario. A linear trendline (dashed) is shown for visualization. Spearman correlation (ρ) and *p*-value express the degree of alignment between Gemini and specialist assessments.

**Figure 6:**
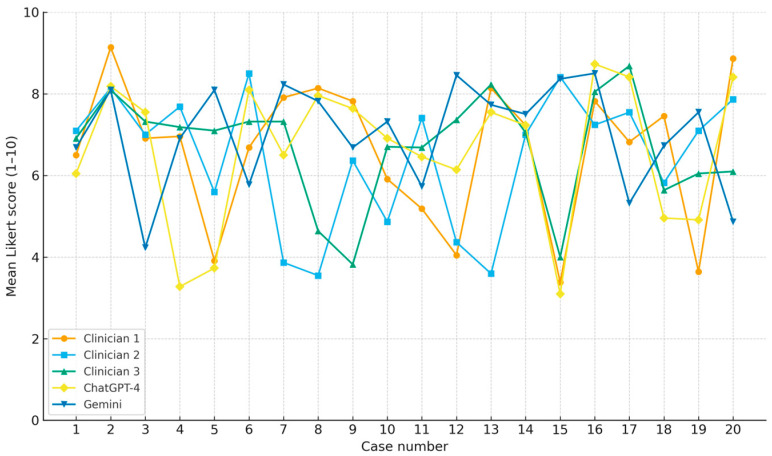
Case-level Likert scores (1–10) for each responder—three maternal–fetal medicine specialists, ChatGPT-4, and Gemini—plotted across 20 clinical scenarios. Distinct markers and colors are used for clarity. The figure illustrates score variability by case and highlights differences in consistency and performance between human and AI responders. Finally, we performed a sub-division to detect straightforward cases such as induction for well-controlled gestational diabetes, as compared with more complex cases, such as managing twin pregnancies with growth discordance; we observed a more grouped agreement between the various responders, including the AI. This can be seen in this figure, where all the cases are presented and average scores of the physicians and AI models are presented altogether. In the straightforward cases, all scores are more tightly grouped, while in the more complex cases, they are less grouped and the AI models are not necessarily located close to the human physicians. Importantly, no AI-generated recommendation was considered clinically unsafe or discordant with established obstetric guidelines according to evaluator review.

For the primary outcome, ChatGPT-4 achieved a mean agreement score of 6.6 (±2.95), with moderate correlation to physician recommendations (ρ = 0.408, *p* < 0.001) and moderate inter-rater reliability (κ = 0.232, *p* < 0.001). Gemini achieved a slightly higher mean score of 7.0 (±2.64) but demonstrated weak negative correlation with physician ratings (ρ = −0.094, *p* = 0.022) and a lack of meaningful inter-rater agreement (κ = −0.024, *p* = 0.352). This pattern reflects that Gemini responses sometimes received relatively high scores overall, but the distribution of its case-specific ratings diverged from the pattern of clinician ratings, resulting in a weak negative case-level correlation. In addition to agreement ratings, evaluators also scored accuracy. ChatGPT-4 achieved similar accuracy to physicians (Wilcoxon *p* = 0.18), while Gemini demonstrated significantly lower accuracy (*p* < 0.01). Figure 2 and Figure 3 illustrate the distribution of evaluator agreement and accuracy scores for ChatGPT-4 and Gemini, respectively. Each histogram reflects all 440 evaluator–case ratings using the 1–10 Likert scale, demonstrating the overall scoring patterns and variability for each model. Figure 4 and Figure 5 illustrate the degree of correlation between AI and human scores, where steeper slopes denote stronger concordance. Figure 6 presents combined mean agreement scores across straightforward vs. complex scenarios. When performing a sub-analysis for cases based on complexity, straightforward versus complex, evaluators showed more consistency in straightforward cases, whereas variability increased substantially in complex scenarios (see Figure 6).

## 5. Discussion

Principal Findings: This study demonstrates that ChatGPT-4’s case-management suggestions in maternal–fetal medicine achieved fair-to-moderate agreement with board-certified specialists (ρ = 0.408; κ = 0.232), particularly in straightforward, guideline-based scenarios. Gemini’s outputs showed weaker and more variable concordance. These results emphasize that while AI Large Language Models can handle and provide solid, effective, and reliable recommendations for routine cases. While disagreement was more pronounced in complex scenarios such as twin–twin transfusion syndrome, suspected placenta accreta requiring peripartum planning, and alloimmunization with anti-Kell, where evaluators often differed in weighting maternal vs. fetal risks and in preferred sequencing of interventions, areas which these modules have yet to master.

Results in the Context of What is Known: The current findings of this study align with what was already previously identified and reported suggesting that Large Language Models such as ChatGPT-4 perform well in structured and straightforward medical tasks [15,16] but still face challenges in scenarios requiring deep clinical reasoning or interpretation [17,18]. Unlike similar and previously conducted studies that evaluated AI in general medical fields, the current study was designed to specifically assess such capabilities in the complex field of maternal–fetal medicine, where the need for precision and context-sensitive recommendations is critical. The limited performance of Gemini compared to ChatGPT-4 underscores that the currently available models show variability in their capabilities, even among advanced models. This variability reinforces the importance of comparing AI tools within specific medical contexts to determine their strengths and limitations.

Unlike outcome-based diagnostic studies, this investigation assessed cognitive alignment between AI models and clinicians. The absence of a clinical ground-truth limits claims about real-world accuracy but allows controlled evaluation of reasoning patterns. This design isolates interpretive quality rather than patient outcomes and should be viewed as an exploratory proof-of-concept.

By focusing specifically on maternal–fetal medicine and using blinded expert evaluations of AI-generated case management decisions, our study addresses the gap identified in prior general AI evaluations, which have rarely examined specialty-specific decision-making in high-risk dual-patient contexts.

Clinical Implications: The moderate alignment of ChatGPT-4’s responses with physician recommendations suggests their potential utility as a supportive tool in clinical settings, especially for routine decision-making or as a second opinion. In practice, ChatGPT-4 could be integrated as a supportive tool for generating preliminary management plans, summarizing guideline-based recommendations, or offering structured checklists for routine scenarios, with final decisions always verified and adapted by MFM specialists. Integration into electronic health records as a decision-support module, with clearly defined guardrails and oversight, may help standardize routine counseling while preserving clinician authority. However, its inconsistent performance in complex scenarios highlights the continuing importance of retaining clinician oversight to ensure patient safety and optimal outcomes.

Research Implications: This study identifies a clear need for further research to enhance the ability to prioritize competing maternal and fetal risks, integrate multiple comorbidities across time, and explicitly articulate alternative management pathways and contingency plans, rather than providing a single, static recommendation. Future investigations should explore strategies to integrate patient-specific data, refine clinical reasoning algorithms, and improve AI performance in diverse medical contexts. Longitudinal studies evaluating the impact of AI-assisted decision-making on patient outcomes could also provide valuable insights.

Strengths and Limitations: A key strength of this study is its focus on a specialized field of medicine, offering a nuanced evaluation of AI capabilities in maternal–fetal medicine. The blinded evaluation methodology minimized potential sources of bias. The relatively high number of specialists and time allocated for the evaluations of the scenarios and the use of multiple AI models provided a comparative perspective. The focus on hypothetical scenarios rather than real-world clinical cases with known outcomes may be a potential caveat. Furthermore, as these scenarios were hypothetical and not linked to patient outcomes, the external validity of our findings is limited. Moreover, we must consider the potential for AI’s hallucinations, plausible but factually incorrect statements, and reliance on outdated or non-peer-reviewed training data. The use of hypothetical cases intentionally eliminated patient-specific confounders and ethical constraints but precluded comparison against actual outcomes. Evaluators lacked access to full contextual data (e.g., longitudinal records, imaging reports, bedside nuances), which may reduce ecological validity and could have led to under- or over-estimation of perceived accuracy compared with real-world decision-making. However, this restriction was intentional to preserve blinding integrity and to ensure that ratings reflected the content and reasoning in the written responses themselves. Only three human clinicians were included to maintain feasibility; their expertise, however, ensures representation of senior decision-making.

These risks further highlight the need for clinician oversight. Additionally, the variability in AI models’ performance highlights the need for standardization and quality control in AI applications. Another limitation is that the 22 evaluators, while all board-certified MFMs, do not represent an absolute gold standard. Their opinions reflect expert practice patterns but may not equate to universally accepted standards of care. Finally, transparency was enhanced by providing the full prompts used to query ChatGPT-4 and Gemini (Appendix A), facilitating replication and model benchmarking in future research.

## 6. Conclusions

AI tools like ChatGPT-4 exhibit a promising potential as supportive aids in maternal–fetal medicine, particularly for routine decision-making. However, their limitations in complex case management necessitate further development and cautious integration into clinical workflows, including rigorous local validation, explicit institutional guidelines for use, and mandatory human oversight of all AI-assisted decisions. Future studies should extend this work to real-world maternal–fetal medicine encounters with prospectively collected outcomes, enabling assessment of both cognitive alignment and actual clinical impact on maternal and neonatal health.

Clinical cases list:

Case 1—Management of oligohydramnios at term.

Case 2—Management of small for gestational age fetus with normal Doppler close to term.

Case 3—Management of well-controlled gestational hypertension.

Case 4—Management for preterm premature rupture of membrane with no signs of chorioamnionitis.

Case 5—Management of intrahepatic cholestasis of pregnancy close to term.

Case 6—Management of mode of delivery and time of delivery in Mono-Chorionic Di-Amniotic (MCDA) twins.

Case 7—Management of well-controlled Gestational Diabetes Mellitus (GDMA1).

Case 8—Management of GDMA1 pregnancy with a large for gestation fetus.

Case 9—Management of TOLAC patient with current macrosomic fetus and previous successful VBACs.

Case 10—Management of twin pregnancy with cervical shortening.

Case 11—Management of Factor 5 Leiden heterozygous mutation, preterm delivery asking for recommendation for future pregnancy.

Case 12—Management of suspected placenta accreta, following three cesarean sections with regard to the usage of intra-aortic balloon.

Case 13—Management of Twin pregnancy di-chorionic di-amniotic, with discordancy and now asking for mode of delivery.

Case 14—Management of mode of delivery after vacuum extraction and 3B perineal tear.

Case 15—Management of late corticosteroids in patient with cervical shortening.

Case 16—Management of medical treatment for early pregnancy CMV seroconversion.

Case 17—Management of TTTS in Mono-Chorionic Di-Amniotic (MCDA) twins.

Case 18—Management of allo-immunization with anti-Kell and increased MCA PSV.

Case 19—Management of cesarean section in 26-week fetus with growth restriction and frank breach.

Case 20—Management of early preterm labor and usage of magnesium sulphate.

## Data Availability

All de-identified scenarios and evaluator ratings are available from the corresponding author upon reasonable request.

## Supplementary Materials

The following supporting information can be downloaded at: https://www.mdpi.com/article/10.3390/jcm15010117/s1, Supplementary S1—Cases & Case management suggestions.

## Appendix A. Prompts Used to Generate AI Responses

“As a Maternal-Fetal Medicine specialist, provide evidence-based management for [case] in ≤3 sentences.”

All prompts entered in new sessions; no prior context retained.
