# Supplementary material for: AI Decision-Making Performance in Maternal–Fetal Medicine: Comparison of ChatGPT-4, Gemini, and Human Specialists in a Cross-Sectional Case-Based Study
Source: J Clin Med. 2025 Dec 24;15(1):117. doi: 10.3390/jcm15010117 (PMC12787038; doi:10.3390/jcm15010117)
Supplement: Supplementary file 1 [file jcm-15-00117-s001.zip › jcm-4038792-supplementary.pdf]

Supplementary S1 – Cases & Case management suggestions

Case 1: A 28-Year-old primipara, 38-weeks and 5 days gestation, comes in for a routine checkup. There are no maternal complications.. Amniotic fluid index (AFI) is measured 4.5 cm. Estimated Fetal Wight (EFW) is 3100 grams. The fetus has a reassuring non-stress test (NST). Would you recommend induction of labor?

Reply a: I would recommend induction of labor. The combination of advanced gestational age—nearly 39 weeks—and a slight indication of placental dysfunction justifies delivery in my opinion.

1. How would you rate the case management (1 -do not agree -10 - agree very much)?

   ____

Reply b: Given the patient's gestational age of 38 weeks and 5 days, normal NST, and reassuring fetal status, I would not recommend induction of labor at this time. However, I would discuss the risks and benefits of expectant management versus induction with the patient, including the likelihood of spontaneous labor within the next week. It is important to note that the decision to induce labor is a complex one that should be made on an individual basis, taking into account the specific circumstances of each patient.

1. How would you rate the case management (1 -do not agree -10 - agree very much)?

   ____

Reply c: Yes, I would recommend induction of labor in this case. The low amniotic fluid index (AFI) of 4.5 cm meets the diagnostic criteria for oligohydramnios (AFI <5 cm), which increases the risk of perinatal complications, including umbilical cord compression and placental insufficiency. Although the fetus shows a reassuring NST result, the reduced amniotic fluid necessitates proactive management to mitigate potential risks. Induction of labor at 38 weeks and 5 days is an appropriate course of action to ensure the safety of both the mother and the baby.

1. How would you rate the case management (1 -do not agree -10 - agree very much)?

   ____

Reply d: Scientific data confirms that even low-risk pregnancies can be safely delivered at 39 weeks, reducing obstetric risks. Low amniotic fluid is certainly a reason for delivery close to 39 weeks. This is due to the complications that low amniotic fluid might indicate (e.g., placental insufficiency) or cause (e.g., cord compression).

1. How would you rate the case management (1 -do not agree -10 - agree very much)?

   ____

Replay e: I would recommend waiting until 39 weeks for induction of labor. Amniotic fluid index (AFI) is a dynamic measurement and can vary within a short time. Although it is currently

on the lower end of the normal range, induction at 39 weeks (in another two days) is supported by the ARRIVE trial, balancing the risks of prolonged pregnancy and potential complications.

1. How would you rate the case management (1 -do not agree -10 - agree very much)?

   ____

Case 2: 24-year-old G1, 37-week gestation with an estimated fetal weight in the 7th percentile. Doppler studies show normal Umbilical Artery (UA) and Middle Cerebral Artery (MCA) blood flow. The mother is otherwise healthy, and the biophysical profile (BPP) score is 8/8. EFW percentiles have been stable (5-10th percentile) since 30 weeks gestation. The patient and her spouse are of average height. Should labor be induced?

Replay a: Not yet. Since growth is consistent and Doppler studies, fetal monitoring, and the biophysical profile are normal, I would continue conservative management at least up until 38 weeks.

1. How would you rate the case management (1 -do not agree -10 - agree very much)?

   ____

Replay b: I would not deliver at 37 weeks. I would wait until 39 weeks and then deliver. A stable 7th percentile with normal Doppler and amniotic fluid levels can benefit from two additional weeks in utero to achieve full maturity. During these two weeks, I would continue with twice-weekly maternal and fetal monitoring.

1. How would you rate the case management (1 -do not agree -10 - agree very much)?

   ____

Replay c: Given the stable fetal growth pattern, normal Doppler studies, and reassuring BPP, expectant management until spontaneous labor or 39 weeks gestation can be considered. While the estimated fetal weight is on the lower side, the stable growth trajectory and normal Doppler studies suggest adequate fetal perfusion.

1. How would you rate the case management (1 -do not agree -10 - agree very much)?

   ____

Replay d: I would not recommend induction at this point. Delivery can be postponed to 38-39 weeks (before 39+6) with close monitoring. Should any signs of fetal compromise arise, induction or cesarean delivery should be promptly initiated.

1. How would you rate the case management (1 -do not agree -10 - agree very much)?

   ____

Replay e: No, labor induction is not recommended at this time. This scenario describes a case of constitutionally small but healthy fetal growth, given the normal Doppler studies, stable growth trajectory, and reassuring biophysical profile (BPP). Without evidence of growth

restriction due to placental insufficiency (e.g., abnormal UA Doppler, suboptimal interval growth, or compromised fetal well-being), there is no indication to induce labor at 37 weeks. Continued close surveillance with weekly Doppler studies, NSTs, and serial ultrasounds is a reasonable approach to ensure ongoing fetal health.

1. How would you rate the case management (1 -do not agree -10 - agree very much)? ______________________

Case 3: A 29-year-old primigravida at 37 weeks with Gestional Hypertension (GHTN) and a favorable cervix (Bishop score >6). Blood pressure is controlled without medication, and fetal growth and well-being are normal. If the patient remains normotensive without signs of preeclampsia, should an induction be pursued now or could expectant management be considered until 39 weeks?

Replay a: Given the gestational hypertension and the favorable cervix, induction of labor can be considered. This would help to reduce the risks associated with gestational hypertension, such as preeclampsia and preterm birth. However, if the patient is stable and there are no signs of worsening disease, expectant management until 39 weeks could be considered, especially if the patient prefers to wait.

1. How would you rate the case management (1 -do not agree -10 - agree very much)?

   ____

Replay b: I recommend induction of labor at 37 weeks, up to 37+6, to mitigate the risk of preeclampsia. The HYPITAT trial demonstrates reduced maternal and neonatal complications with timely induction, without increasing neonatal morbidity.

1. How would you rate the case management (1 -do not agree -10 - agree very much)?

   ____

Replay c: Due to the risk of progression to preeclampsia with severe features and the potential complications, I recommend induction of labor, especially since cervical conditions (Bishop score) indicate a good chance of a successful induction.

1. How would you rate the case management (1 -do not agree -10 - agree very much)?

   ____

Replay d: Induction of labor should be pursued now. GHTN at 37 weeks is associated with an increased risk of maternal and fetal complications, such as preeclampsia and placental insufficiency, even if blood pressure is currently controlled. Current evidence, including ACOG recommendations, support delivery at 37 weeks for GHTN to optimize maternal and fetal outcomes. With a favorable Bishop score, induction is likely to proceed successfully, minimizing risks for both the mother and baby.

1. How would you rate the case management (1 -do not agree -10 - agree very much)?

   ____

Replay e: I would recommend delivery. The risks of GHTN at37 weeks in terms of maternal complications, including progression to severe preeclampsia, outweigh the potential benefits of improving fetal maturity by prolonging the pregnancy.

1. How would you rate the case management (1 -do not agree -10 - agree very much)?

   ____

Case 4: A 35-week and 4 days primigravida presents with Premature Rupture of Membrane (PROM), with no signs of chorioamnionitis or labor. The fetal heart rate and the mother's vitals are stable, lung maturation has been addressed with late corticosteroids. Should labor be induced to avoid prolonged rupture risks, or could conservative management be extended with close monitoring to avoid prematurity-related complications?

Replay a: Given the premature rupture of membranes (PROM) and the risk of chorioamnionitis, induction of labor should be considered. However, the decision to induce should be made on a case-by-case basis, taking into account the specific circumstances of each patient. If the patient is stable and there are no signs of infection, conservative management with close monitoring may be considered for a short period.

1. How would you rate the case management (1 -do not agree -10 - agree very much)?

   ____

Replay b: Labor should be induced. Expectant management in PROM after 34 weeks increases the risk of infection (chorioamnionitis and neonatal sepsis) without significantly reducing neonatal morbidity related to prematurity. With stable maternal and fetal status, after completing antenatal corticosteroids, induction at 35+4 weeks gestation is the safer approach to avoid the complications of prolonged rupture.

1. How would you rate the case management (1 -do not agree -10 - agree very much)?

   ____

Replay c: Continue conservative management. Conservative management at this gestational age has shown neonatal benefits with low maternal risk. Therefore, under strict monitoring, I prefer conservative management.

1. How would you rate the case management (1 -do not agree -10 - agree very much)?

   ____

Replay d: Conservative management can be offered up to 36 weeks, emphasizing shared decision-making with the patient. Risks of infection should be clearly explained, but spontaneous labor often occurs naturally in these cases and should not be suppressed with tocolytics.

1. How would you rate the case management (1 -do not agree -10 - agree very much)?

   ____

Replay e: I recommend close conservative follow-up until 36 weeks to allow for further lung maturity, provided there are no clinical or laboratory signs of infection.

1. How would you rate the case management (1 -do not agree -10 - agree very much)?

   ____

Case 5: A 36-week pregnant patient with mild intrahepatic cholestasis of pregnancy ICP (bile acids of 12 μmol/L, with controlled itching) and no signs of fetal distress. Should induction be considered at 37 weeks or could expectant management continue with close fetal surveillance?

Replay a: Induction at 37 weeks is recommended. ICP is associated with an increased risk of stillbirth, especially with bile acids >10 μmol/L. While fetal surveillance is reassuring, delivery at 37 weeks minimizes potential risks without adding significant neonatal complications due to prematurity.

1. How would you rate the case management (1 -do not agree -10 - agree very much)?

   ____

Replay b: I recommend induction of labor at 37 weeks due to the unpredictable effects of bile acids on fetal heart function.

1. How would you rate the case management (1 -do not agree -10 - agree very much)?

   ____

Replay c: Expectant management is reasonable. For non-severe ICP (bile acids <100 μmol/L), induction at a later gestational age decreases the risk of neonatal complications while minimizing the risk of intrauterine fetal demise (IUFD).

1. How would you rate the case management (1 -do not agree -10 - agree very much)?

   ____

Replay d: Recommend delivery at 37 to 38 weeks due to the risk of intrauterine fetal demise, even if very rare.

1. How would you rate the case management (1 -do not agree -10 - agree very much)?

   ____

Replay e: Given the mild ICP and the absence of fetal distress, expectant management with close fetal surveillance can be considered. Induction of labor at 37 weeks could be considered if there is a significant worsening of ICP symptoms or fetal distress. However, the decision to induce should be made on a case-by-case basis, taking into account the specific circumstances of each patient.

1. How would you rate the case management (1 -do not agree -10 - agree very much)?

   ____

Case 6: At 36 weeks, A patient presents with Mono-Chorionic Di-Amniotic (MCDA) twins with a 15% growth discordance. Both fetuses have normal Doppler studies and no signs of Twin To Twin Transfusion Syndrome (TTTS) or Twin Anemia Polycythemia Syndrome (TAPS). Should induction or caesarean section be performed now or would expectant management with daily monitoring provide safer outcomes?

Replay a: 36 gestation weeks is the average delivery time for twins, and placental complications in monochorionic twins tend to occur earlier than in singleton or dichorionic twin pregnancies. Therefore, I recommend delivery at this time.

1. How would you rate the case management (1 -do not agree -10 - agree very much)?

   ____

Replay b: Delivery is recommended between 36 and 37 weeks. Beyond 37 weeks, I would not advocate conservative management due to increased maternal and fetal risks.

1. How would you rate the case management (1 -do not agree -10 - agree very much)?

   ____

Replay c: Delivery at 36-37 weeks is recommended. While fetal well-being is currently reassuring, MCDA twins are at higher risk of sudden deterioration, including TTTS or TAPS. Elective delivery (induction or cesarean, depending on fetal lie) at 36-37 weeks balances the risks of prematurity and the complications associated with prolonged gestation.

1. How would you rate the case management (1 -do not agree -10 - agree very much)?

   ____

Replay d: Delivery should be planned between 36-37 weeks, provided there are no additional complications. A 15% growth discordance is within normal limits, as selective intrauterine growth restriction (SIUGR) is typically defined by a discordance >25%.

1. How would you rate the case management (1 -do not agree -10 - agree very much)?

   ____

Replay e: Given the stable fetal status and absence of complications, expectant management with daily monitoring may be considered. However, the decision should be individualized and based on the specific circumstances of the pregnancy. If there are any signs of worsening fetal condition or preterm labor, induction or cesarean section may be necessary.

1. How would you rate the case management (1 -do not agree -10 - agree very much)?

   ____

Case 7: A patient presents at 38-weeks gestation with well-controlled gestational diabetes (GDMA1). The fetus measures in the 80th percentile, with an estimated fetal weight ofg 3700 grams. Should labor be induced or is it better to wait for spontaneous labor?

Replay a: Delivery can be postponed to a maximum of 39 weeks. This approach is consistent with ACOG and RCOG guidelines, as the fetus is not macrosomic and the maternal pelvis appears adequate. If concerns about fetopelvic disproportion arise, reevaluation may be warranted.

1. How would you rate the case management (1 -do not agree -10 - agree very much)?

   ____

Replay b: Expectant management with close monitoring is reasonable. The fetus is appropriately grown, and there are no complications warranting induction. Waiting for spontaneous labor at term while monitoring for signs of fetal distress or macrosomia is an acceptable course.

1. How would you rate the case management (1 -do not agree -10 - agree very much)?

   ____

Relpay c: Given the well-controlled gestational diabetes and the estimated fetal weight, expectant management with close fetal monitoring may be considered. However, the decision to induce or allow spontaneous labor should be made on a case-by-case basis, taking into account the specific circumstances of the pregnancy.

1. How would you rate the case management (1 -do not agree -10 - agree very much)?

   ____

Replay d: If diabetes is well-controlled and the estimated weight is below 4000g, it’s better to wait until 39 weeks. At39 weeks, lung maturity and induction success rates are better. I recommend induction at 39 weeks if spontaneous labor does not occur (meanwhile, consider membrane stripping if possible).

1. How would you rate the case management (1 -do not agree -10 - agree very much)?

   ____

Replay e: I would recommend induction of labor due to suspected impending macrosomia. Based on a Lancet study showing lower rates of shoulder dystocia, I support this approach.

1. How would you rate the case management (1 -do not agree -10 - agree very much)?

   ____

Case 8: A 32 y/o patient presents to triage on the 38th week of her second pregnancy. Her previous pregnancy was normal with no diabetes, and she delivered a 3800gram baby at term,with no complications. This pregnancy she has GDMA1 with glucose levels well controlled without medication. Estimated Fetal Wight (EFW) is 4050 grams. Normal Amniotic fluid index (AFI) and Non-Stress Test (NST). Pelvis is adequate and patient wants to avoid a caesarean section (CS). Will you recommend a caesarean section (CS) or attempt vaginal delivery?

Replay a: The birth weight of the first child gives confidence regarding the ability of the pelvis to handle delivery. The current estimated weight is suitable for vaginal delivery if the diabetes is controlled (weight estimate below 4250g). I would explain the margin of error in weight estimation and the risk of shoulder dystocia. If the patient insists, I would proceed with vaginal delivery.

1. How would you rate the case management (1 -do not agree -10 - agree very much)?

   ____

Replay b: I recommend attempting vaginal delivery. Cesarean delivery should be considered if the estimated fetal weight exceeds 4.5 kg in patients with GDM. A thorough discussion about the risk of shoulder dystocia should be conducted.

1. How would you rate the case management (1 -do not agree -10 - agree very much)?

   ____

Replay c: Given the estimated fetal weight and the patient's desire to avoid a cesarean section, a trial of labor may be considered. However, the decision to proceed with a vaginal delivery should be made carefully, taking into account the potential risks of shoulder dystocia and other complications associated with large babies. If there are any concerns about fetal well-being or other complications, a cesarean section may be recommended.

1. How would you rate the case management (1 -do not agree -10 - agree very much)?

   ____

Replay d: Recommend cesarean section. This is due to the increased risk of shoulder dystocia given the patient's diabetes and the macrosomic estimated fetal weight.

1. How would you rate the case management (1 -do not agree -10 - agree very much)?

   ____

Replay e: Attempt vaginal delivery. With a favorable obstetric history and maternal preference to avoid cesarean delivery, a trial of labor is appropriate, particularly given the controlled GDM and adequate pelvis. However, careful monitoring for labor progress and signs of shoulder dystocia is essential.

1. How would you rate the case management (1 -do not agree -10 - agree very much)?

   ____

Case 9: A patient with a history of one prior caesarean section (CS) presents at 39 weeks gestation with suspected macrosomia (estimated fetal weight of 4,100 grams). This is her 4th delivery. The first was a caesarean section (CS) for breech lie and the 2nd and 3rd were Vaginal Births After a caesarean section (VBACs) at 3300 and 3400 grams. The patient desires a trial of labor after caesarean (TOLAC) and to avoid a second caesarean section (CS). Should an elective caesarean section (CS) be scheduled, or could atrial of labor after caesarean (TOLAC) be justified?

Replay a: Given the patient's history of previous cesarean sections and the estimated fetal weight, an elective cesarean section may be the safest option. However, if the patient is determined to have a trial of labor, a careful assessment of the risks and benefits should be performed. A TOLAC should only be attempted in a hospital setting with appropriate resources and personnel.

1. How would you rate the case management (1 -do not agree -10 - agree very much)?

   ____

Replay b: Attempting vaginal delivery can certainly be justified. The most important factor for predicting success—a history of vaginal births—is present, and the reason for the first cesarean is also a positive prognostic factor. While the estimated weight of 4100g lowers the chances of success (especially compared to the lower weights of prior births), it is reasonable to respect her desire to attempt labor.

1. How would you rate the case management (1 -do not agree -10 - agree very much)?

   ____

Replay c: TOLAC is reasonable if the patient desires. The patient has a history of successful VBACs, making her a good candidate for TOLAC despite suspected macrosomia. Close intrapartum monitoring is essential, with a low threshold for cesarean delivery if complications arise.

1. How would you rate the case management (1 -do not agree -10 - agree very much)?

   ____

Replay d: I recommend cesarean delivery in this case. For patients undergoing a trial of labor after cesarean (TOLAC), additional risk factors such as suspected macrosomia increase the risk of uterine rupture, particularly at full dilation.

1. How would you rate the case management (1 -do not agree -10 - agree very much)?

   ____

Replay e: I would recommend vaginal delivery after discussing all the risks associated with attempting labor in this situation.

1. How would you rate the case management (1 -do not agree -10 - agree very much)?

   ____

Case 10: A patient with a twin pregnancy presents to triage at 23 weeks gestation. This is her first pregnancy. A routine anatomy scan she performed that day, demonstrated, a cervical shortening to 15mm with extensive funneling. No previous relevant medical history. No signs of infection. Pelvic exam demonstrates a closed firm, mildly shortened cervix. Will you recommend a cerclage or manage with progesterone and surveillance?

Replay a: The data on cervical cerclage in twins is not more convincing than the data on progesterone (for cervical shortening without dilation). Additionally, there is evidence of poorer outcomes with cerclage in twin pregnancies. Therefore, in this case, I recommend progesterone therapy rather than cerclage.

1. How would you rate the case management (1 -do not agree -10 - agree very much)?

   ____

Replay b: I would recommend cervical cerclage. Although evidence for cerclage efficacy in cases of isolated cervical shortening in twins is limited, clear evidence supports its efficacy in cases of cervical dilation. At 23 weeks, conservative management and reassessment are limited.

1. How would you rate the case management (1 -do not agree -10 - agree very much)?

   ____

Replay c: Given the short cervix and funneling, a cerclage may be considered to help prevent preterm birth. However, the decision to perform a cerclage should be made on a case-by-case basis, taking into account the specific circumstances of the pregnancy. Progesterone supplementation may also be considered as an adjunct therapy.

1. How would you rate the case management (1 -do not agree -10 - agree very much)?

   ____

Replay d: Cerclage is recommended. With significant cervical shortening (<15 mm) and no signs of infection, a cerclage is appropriate for twin pregnancies in the second trimester to reduce the risk of preterm birth. Progesterone supplementation and close surveillance should accompany the intervention.

1. How would you rate the case management (1 -do not agree -10 - agree very much)?

   ____

Replay e: A cerclage is recommended. Evidence suggests improved outcomes in twin pregnancies with cervical length <15 mm when cerclage is performed, as demonstrated in studies such as Roman et al.

1. How would you rate the case management (1 -do not agree -10 - agree very much)?

   ____

Case 11: A primipara with a Factor 5 Leiden heterozygous mutation delivers at 30 weeks getstation due to preeclampsia (PET) with severe features. Baby's wight was in the 3rd percentile and placenta demonstrated extensive signs of malperfusion. Would you recommend Low Molecular Heparin for her next pregnancy?

Replay a: Low-molecular-weight heparin (LMWH) is recommended. Given her history of preeclampsia, severe placental malperfusion, and prematurity, LMWH prophylaxis can reduce risks of thrombosis-related placental insufficiency and recurrence of adverse outcomes in subsequent pregnancies.

1. How would you rate the case management (1 -do not agree -10 - agree very much)?

   ____

Replay b: Given the history of severe preeclampsia and fetal growth restriction, low-molecular-weight heparin (LMWH) should be strongly considered in the next pregnancy. LMWH can help to reduce the risk of blood clots and improve blood flow to the placenta.

1. How would you rate the case management (1 -do not agree -10 - agree very much)?

   ____

Replay c: No. There is no established connection between Factor V Leiden carrier status and placental insufficiency (severe preeclampsia and growth restriction). For the next pregnancy, I would recommend aspirin only, with LMWH limited to the postpartum period for VTE prevention.

1. How would you rate the case management (1 -do not agree -10 - agree very much)?

   ____

Replay d: I would recommend aspirin and low-molecular-weight heparin (LMWH) for the next pregnancy. While I don't routinely treat Factor V Leiden carriers during pregnancy, the presence of placental pathology with maternal vascular malperfusion lesions and sings indicating microthromi warrants adding LMWH to aspirin therapy.

1. How would you rate the case management (1 -do not agree -10 - agree very much)?

   ____

Replay e: I would initiate prophylactic low-molecular-weight heparin (LMWH). While the patient does not meet strict criteria (e.g., homozygous mutation or prior events), the presence of significant vascular issues strongly indicates a benefit from anticoagulation to prevent recurrent thrombotic events.

1. How would you rate the case management (1 -do not agree -10 - agree very much)?

   ____

Case 12: A patient is scheduled for a caesarean section at 35 weeks gestation due to a suspected placenta accreta. She has previously had 3 caesarean sections. In this pregnancy, ultrasound scans demonstrate placenta previa with a high index of suspicion for placenta accreta. The plan is to perform a cesarean hysterectomy at 35 weeks gestation. Will you recommend preparation with insertion of arterial balloons under invasive radiology as part of the preparation for surgery?

Replay a: No. The literature does not definitively support embolization benefits in accreta cases.

1. How would you rate the case management (1 -do not agree -10 - agree very much)?

   ____

Replay b: Yes, arterial balloon catheter placement is recommended. Preoperative placement of arterial balloons allows for targeted control of blood flow during surgery, reducing hemorrhage risk. This technique, combined with a multidisciplinary approach, is considered standard preparation for suspected placenta accreta cases.

1. How would you rate the case management (1 -do not agree -10 - agree very much)?

   ____

Replay c: No. If the plan already includes hysterectomy, I would not recommend inserting arterial catheters into the iliac arteries unless there is an intention to preserve the uterus (to reduce bleeding during placental separation attempts).

1. How would you rate the case management (1 -do not agree -10 - agree very much)?

   ____

Replay d: Insertion of arterial balloons under invasive radiology can be a valuable tool to reduce blood loss during cesarean hysterectomy for placenta accreta. The decision to use this technique should be made on a case-by-case basis, taking into account the specific circumstances of the patient.

1. How would you rate the case management (1 -do not agree -10 - agree very much)?

   ____

Replay e: If arterial balloons are available at the center, I would recommend their use. Balloon placement can effectively minimize blood loss during surgery, providing a safer operative course in cases of placenta accreta.

1. How would you rate the case management (1 -do not agree -10 - agree very much)?

   ____

Case 13: A primipara with a Di-Chorionic Di-Amniotic twin pregnancy, arrives at 36 gestational weeks to discuss mode of delivery. The pregnancy has been completely normal. Fetuses are vertex/vertex.. The Estimated fetal weight (EFW) for twin A is 2010 grams and the estimated fetal weight (EFW) for twin B is 2879 grams. Will you recommend a vaginal delivery or schedule a caesarean section?

Replay a: A vaginal delivery is recommended. With both twins in vertex presentation, after a normal pregnancy progression, and no contraindications, vaginal delivery is appropriate. It offers lower maternal morbidity compared to cesarean section, provided that continuous monitoring and an experienced team are available to handle twin delivery.

1. How would you rate the case management (1 -do not agree -10 - agree very much)?

   ____

Replay b: I recommend a cesarean section due to the 30% fetal weight discordance and the risk of head entrapment for twin B in the case of breech presentation of the second twin.

1. How would you rate the case management (1 -do not agree -10 - agree very much)?

   ____

Replay c: Despite the 30% fetal weight discordance, I recommend vaginal delivery when both fetuses are initially in vertex presentation. The mother and team should be prepared for cesarean delivery of the second twin if it changes to a non-vertex position after the first twin is delivered.

1. How would you rate the case management (1 -do not agree -10 - agree very much)?

   ____

Replay d: Vaginal delivery is a good option if both twins are vertex, as it avoids a primary cesarean section in this primiparous patient. However, it is important to counsel the patient about the possibility of the second twin turning to a breech lie, in which case breech extraction would not be recommended due to the 30% weight discordance.

1. How would you rate the case management (1 -do not agree -10 - agree very much)?

   ____

Replay e: Given the normal fetal growth and well-being, a vaginal delivery may be considered. However, the decision should be made on a case-by-case basis, taking into account the specific circumstances of the pregnancy. If there are any concerns about fetal well-being or other complications, a cesarean section may be recommended.

1. How would you rate the case management (1 -do not agree -10 - agree very much)?

   ____

Case 14: A Patient presents for counseling in her second pregnancy. In her previous pregnancy she delivered at 38 weeks gestation a 3700 gram baby by vacuum extraction (VE) and had a 3B perineal tear that was repaired. She is asymptomatic. Manometry is normal but a transrectal ultrasound (TRUS) demonstrates scar tissue at 2 different sites in the anal sphincter. The patient desires a vaginal delivery. Would you recommend a Cesarean or vaginal delivery?

Replay a: Due to the potential of devastating effects on quality of life from advanced tears and due to the scars visible on sphincter imaging, and particularly because she healed well and is asymptomatic now, I would not want to challenge her sphincter with a vaginal delivery. I recommend an elective cesarean section.

1. How would you rate the case management (1 -do not agree -10 - agree very much)?

   ____

Replay b: Cesarean delivery is recommended. Despite the patient's preference, a history of a 3B perineal tear with scar tissue poses a significant risk for recurrence and worsened pelvic floor damage with subsequent vaginal delivery. Cesarean section is the safer choice to prevent anal incontinence and further injury.

1. How would you rate the case management (1 -do not agree -10 - agree very much)?

   ____

Replay c: If the estimated fetal weight is similar (or slightly lower), I would recommend a cesarean section. There is approximately a 7% risk of recurrent severe perineal tear, which could significantly impact quality of life.

1. How would you rate the case management (1 -do not agree -10 - agree very much)?

   ____

Replay d: Given the history of perineal trauma and anal sphincter injury, a cesarean section may be the safest option to avoid further injury. However, the decision should be made on a case-by-case basis, taking into account the specific circumstances of the patient.

1. How would you rate the case management (1 -do not agree -10 - agree very much)?

   ____

Replay e: Vaginal delivery can be considered if the patient wishes, but she must be counseled on the doubled risk of recurrence and the potential long-term incontinence. Reviewing the estimated fetal weight (EFW) is essential to make a more informed recommendation.

1. How would you rate the case management (1 -do not agree -10 - agree very much)?

   ____

Case 15: A patient presents to triage at 34+4 gestational weeks with contractions. Cervix is measured at 15 mm and dilated, 80% effaced and soft. After IV fluids and surveillance the contractions resolve. No signs of infection. Fetal wellbeing with ultrasound and Non-Stress Test (NST) is normal. You decide to admit her to the ward. Will you administer a course of late corticosteroids?

Replay a: Given the resolved contractions and stable cervical examination, a course of late corticosteroids may not be necessary. However, the decision should be made on a case-by-case basis, taking into account the specific circumstances of the pregnancy.

1. How would you rate the case management (1 -do not agree -10 - agree very much)?

   ____

Replay b: Yes, I would administer late corticosteroids.

1. How would you rate the case management (1 -do not agree -10 - agree very much)?

   ____

Replay c: If it is only a cervical shortening to 15 mm without any dilation, the contractions have ceased, and I assess a low likelihood of delivery within the next week, I would not administer late corticosteroids.

1. How would you rate the case management (1 -do not agree -10 - agree very much)?

   ____

Replay d: No, I would not administer late corticosteroids in this case. Late antenatal corticosteroids are indicated only for cases of iatrogenic preterm delivery or when there is a high risk of cesarean section, which does not apply here.

1. How would you rate the case management (1 -do not agree -10 - agree very much)?

   ____

Replay e: Administer late corticosteroids. A single course of corticosteroids at 34+4 weeks is beneficial to reduce neonatal respiratory morbidity, particularly given cervical changes indicating preterm labor risk. The absence of active labor or infection does not preclude the benefit of this intervention.

1. How would you rate the case management (1 -do not agree -10 - agree very much)?

   ____

Case 16: A patient presents at 8 weeks of pregnancy for a routine obstetric visit. Serologies for Cytomegaly Virus (CMV) return IGG positive IGM positive, Vidas test for IGM positive with low avidity. History reveals a partner who reports severe pharyngitis around the time of conception Will you recommend initiating secondary prevention with Valcyclovir?

Replay a: Yes, I would start treatment promptly to reduce the risk of vertical transmission. Early intervention minimizes complications and improves neonatal outcomes.

1. How would you rate the case management (1 -do not agree -10 - agree very much)?

   ____

Replay b: Given the positive CMV IgM and low avidity, valacyclovir may be considered to reduce the risk of fetal infection. However, the decision to initiate valacyclovir should be made on a case-by-case basis, taking into account the specific circumstances of the pregnancy.

1. How would you rate the case management (1 -do not agree -10 - agree very much)?

   ____

Replay c: Yes, the timing of the infection—likely around conception or very early in pregnancy, as indicated by low avidity—justifies treatment. I recommend valacyclovir, as it has been proven effective in preventing fetal infection during critical developmental stages.

1. How would you rate the case management (1 -do not agree -10 - agree very much)?

   ____

Replay d: Recommend secondary prevention with valacyclovir. The combination of positive IgM, low avidity, and partner's pharyngitis history strongly indicates a primary CMV infection. Valacyclovir can reduce the risk of vertical transmission, particularly during early pregnancy, while close monitoring with serial ultrasounds is crucial.

1. How would you rate the case management (1 -do not agree -10 - agree very much)?

   ____

Replay e: I recommend valacyclovir treatment based on a recent RCT study that demonstrated reduced rates of congenital CMV infection in treated patients.

1. How would you rate the case management (1 -do not agree -10 - agree very much)?

   ____

Case 17: A primipara with monochorionic twins arrives at her high-risk prenatal care at 20 weeks. On ultrasound both fetuses have similar weights. The maximal vertical pocket for Twin A is 11 Cm and for twin B 1.5 cm. Both bladders are visible, Dopplers studies are normal and the pregnancy is otherwise uncomplicated. Will you recommend or expectant management or laser therapy for Twin To Twin Transfusion Syndrome (TTTS) at this point?

Replay a: Given the severe discordance in amniotic fluid volumes, laser ablation of placental vessels may be considered to treat TTTS. However, the decision to perform laser ablation should be made by a specialist in fetal medicine.

1. How would you rate the case management (1 -do not agree -10 - agree very much)?

____

Replay b: I would not perform laser treatment for Stage 1 TTTS. Only 30% of cases progress to Stage 2, while some improve spontaneously. I would continue with conservative monitoring.

1. How would you rate the case management (1 -do not agree -10 - agree very much)?

____

Replay c: Expectant management is appropriate. Twin A has a maximal vertical pocket (MVP) >8 cm, and Twin B <2 cm, consistent with Stage 1 TTTS. However, both bladders are visible, Dopplers are normal, and the condition is stable, justifying close monitoring rather than immediate intervention with laser ablation.

1. How would you rate the case management (1 -do not agree -10 - agree very much)?

____

Replay d: Conservative follow-up is appropriate. Laser treatment should be considered only if the condition progresses to Quintero Stage 2.

1. How would you rate the case management (1 -do not agree -10 - agree very much)?

____

Replay e: Expectant management is appropriate at this stage. Laser therapy is typically reserved for Stage 2 TTTS or higher, within the gestational window of 16 to 26 weeks. Close monitoring is critical.

1. How would you rate the case management (1 -do not agree -10 - agree very much)?

____

Case 18: A patient is closely monitored in a high risk clinic for allo-imunization with anti Kell antibodies with a titer of 1:128. Middle Cerebral Artery Peak Systolic Velocity (MCA PSV) was normal throughout the pregnancy. At 32 weeks MCA PSVwas 1.3 MoM and today at 34+4 weeks it is 1.7 MoM in repeated accurate measurements. Pregnancy is otherwise normal. Will you recommend fetal blood transfusion? delivery or expectant management?

Replay a: Certainly not conservative management. I would deliver at 34+4. By 35 weeks, there's no doubt that delivery is advisable. At 34+4, the benefit of waiting two more days is outweighed by the risks, so I recommend delivery.

1. How would you rate the case management (1 -do not agree -10 - agree very much)?

   ____

Replay b: Intrauterine transfusion (IUT) is recommended if the hematocrit is <30%, and can be performed up to 35 weeks. The goal is to prevent significant anemia and ensure delivery of a healthy baby within 2–3 weeks. The principle "Kell kills" highlights the need for proactive management even with low titers.

1. How would you rate the case management (1 -do not agree -10 - agree very much)?

   ____

Replay c: Given the rising MCA PSV, fetal blood transfusion may be considered to improve fetal oxygenation. However, the decision to perform a fetal blood transfusion should be made on a case-by-case basis, taking into account the specific circumstances of the pregnancy.

1. How would you rate the case management (1 -do not agree -10 - agree very much)?

   ____

Replay d: Fetal blood transfusion is recommended. An MCA PSV ≥1.5 MoM indicates fetal anemia due to alloimmunization. Fetal blood transfusion is the treatment of choice to address anemia and avoid complications, allowing for further prolongation of pregnancy.

1. How would you rate the case management (1 -do not agree -10 - agree very much)?

   ____

Replay e: Severe anemia is suspected. At this stage, I would deliver and provide a transfusion to the newborn. (After 35 weeks, monitoring for anemia becomes unreliable with Dopplers, so an intrauterine transfusion would complicate fetal assessment).

1. How would you rate the case management (1 -do not agree -10 - agree very much)?

   ____

Case 19: A primipara delivers by cesarean section at 26 weeks gestation due to severe preeclampsia and fetal growth restriction (FGR). The estimated fetal weight is 700 grams and it is in a frank breech position. Will you perform the cesarean section in a classic or low transverse incision?

Replay a: Perform a classic cesarean section. With extreme prematurity (26 weeks) and a frank breech presentation, a classic cesarean section ensures adequate access and minimizes risk of trauma to the fetus or uterus, despite slightly higher long-term maternal risks.

1. How would you rate the case management (1 -do not agree -10 - agree very much)? ______________________

Replay b: Classic cesarean section—absolute indication.

1. How would you rate the case management (1 -do not agree -10 - agree very much)? ______________________

Replay c: A classic cesarean section is advised if the lower uterine segment is not developed. This approach ensures a safer delivery and reduces the risk of complications, as the low transverse incision may involve the thicker myometrium and compromise future uterine integrity.

1. How would you rate the case management (1 -do not agree -10 - agree very much)? ______________________

Replay d: I would perform a low transverse incision cesarean section. However, I would not hesitate to extend the uterine incision into a T-shape if necessary.

1. How would you rate the case management (1 -do not agree -10 - agree very much)? ______________________

Replay e: Given the gestational age and fetal presentation, a classic cesarean incision may be necessary to facilitate delivery. However, the decision should be made on a case-by-case basis, taking into account the specific circumstances of the pregnancy.

1. How would you rate the case management (1 -do not agree -10 - agree very much)? ______________________

Case 20: A patient is admitted to the delivery ward at 29 weeks gestation with threatened preterm labor and 3 cm dilatation. Fetus is in vertex presentation. Steroids are administered as well as Atosiban and Magesium Sulfate for neuroprotection. After 8 hours in the delivery ward contractions resolve, cervix is stable, and you decide to stop Magnesium administration and observe. 12 hours later the patient notes vaginal pressure and is found at 8 cm dilatation and preparations for vaginal delivery are initiated. Will you renew the Magnesium Sulphated administration during delivery?

Replay a: Yes, I would renew magnesium sulfate administration during delivery. Since the exact timing of delivery is uncertain, the fetus could benefit neurologically from magnesium.

1. How would you rate the case management (1 -do not agree -10 - agree very much)?

   ____

Replay b: Renew magnesium sulfate administration during delivery. Magnesium sulfate provides neuroprotection for preterm neonates, particularly when delivery appears imminent. Restarting magnesium during delivery ensures maximal benefit, as neuroprotection is most effective within 24 hours before birth.

1. How would you rate the case management (1 -do not agree -10 - agree very much)?

   ____

Replay c: I would certainly restart magnesium sulfate, as the previous dose would no longer be effective after 8 hours. Magnesium's primary neuroprotective effect is around the time of delivery.

1. How would you rate the case management (1 -do not agree -10 - agree very much)?

   ____

Replay d: Given the resolved contractions and stable cervical examination, the magnesium sulfate may be discontinued. However, the decision to restart magnesium sulfate during delivery should be made on a case-by-case basis, taking into account the specific circumstances of the labor.

1. How would you rate the case management (1 -do not agree -10 - agree very much)?

   ____

Replay e: Yes, magnesium sulfate should be administered 20 minutes before birth for neuroprotection. This timing optimizes the benefits, particularly for preterm neonates.

1. How would you rate the case management (1 -do not agree -10 - agree very much)?

   ____
